# Too Close to Speak Up? How Group Network Density and Status Conflict Affect Group Voice

**DOI:** 10.3390/bs15070926

**Published:** 2025-07-09

**Authors:** Yumi Ko, Myung-Ho Chung, Dongwon Choi

**Affiliations:** Ewha School of Business, Ewha Womans University, 52-Ewhayeodae-gil, Seodaemun-gu, Seoul 03760, Republic of Korea; myhoc@ewha.ac.kr (M.-H.C.); dwchoi@ewha.ac.kr (D.C.)

**Keywords:** group voice, social network, group network density, status conflict

## Abstract

Although group network characteristics significantly influence a group’s ability to exchange and absorb knowledge by listening to group members’ opinions, previous research on voice behavior has not yet fully addressed the social and relational factors in work groups that affect group-level voice. Specifically, in line with the “dark side of social capital” argument, this study examined the effects of group network density on group voice. In addition, drawing on the notion of status conflict, we further examined the moderating role of status conflict on the relationship between group network density and group voice. Using data from 55 work groups, we found an inverted U-shaped relationship between group network density and group voice. Moreover, we found that status conflict moderated the inverted-U effect of group density on group voice, such that when status conflict was high, (1) the overall level of group voice was reduced and (2) group voice decreased faster on the downward side of the inverted-U curve. Herein, we discuss the theoretical and practical implications of these results with relation to effective group management.

## 1. Introduction

Group voice behavior refers to the extent to which members of a work group collectively engage in expressing constructive suggestions, raising concerns, or offering feedback aimed at improving team functioning, group outcomes, or organizational effectiveness. While individual voice is typically conceptualized as a discretionary, extra-role behavior performed by a single employee (Van Dyne & LePine, 1998; LePine & Van Dyne, 2001), group voice captures the aggregate, shared, or emergent expression of voice within a social unit, such as a team or work group (Zheng et al., 2022). Group voice behavior is not simply the sum of individual voices. Rather, it reflects the collective orientation toward speaking up, which emerges through group dynamics, social norms, and relational structures. This includes how voice is enacted, encouraged, or suppressed within the specific interpersonal and structural context of the group (Morrison, 2011; Frazier & Bowler, 2015). As such, group voice is inherently contextual, relational, and embedded within social interactions, and is better understood through a collective lens rather than purely individual psychological characteristics.

The extant research has overly focused on individual-level voice; only a few studies have investigated employee voice as a collective-level phenomenon. However, there are several key distinctions that necessitate treating group voice as a separate and important construct in organizational research. First, while individual voice may occur as a private act between an employee and a supervisor, group voice is a public act, visible to multiple group members. This visibility raises the social costs and benefits of speaking up, making group voice more sensitive to power dynamics, status hierarchies, and network structures (Zerubavel, 2006; Bendersky & Hays, 2012). Also, while investigation of individual-level voice allows us to figure out whether employees speak or not, it does not necessarily lead to active communication among all group members. For instance, while the majority of the group members may raise their voices, minority members may keep silent, which is not fruitful in facilitating team innovation (De Dreu & West, 2001). Second, individual voice is often driven by personal attributes (e.g., self-efficacy, personality), whereas group voice arises from interpersonal interactions, norms of communication, and the relational climate within a group (Tangirala & Ramanujam, 2008). Third, while individual voice can be overlooked or dismissed, when multiple group members voice concerns collectively, the message becomes more salient, legitimate, and harder to ignore (Frazier & Bowler, 2009; Li et al., 2017). Thus, group voice has a disproportionate impact on team learning, innovation, and performance. Fourth, the risks associated with voice behavior—such as fear of retaliation or damaging relationships—are amplified at the group level, where the audience is broader and more socially significant. This makes group social capital particularly important enablers of group voice (Morrison & Milliken, 2000). Lastly, group-level variables, such as group network density and status conflict, influence the collective propensity to engage in voice. These structural factors are often invisible when focusing solely on individual-level predictors (Burt, 2004; Coleman, 1988).

Studying group voice behavior offers richer insights into how employee input is shaped by social context and collective processes (Sherf et al., 2018; Bain et al., 2021; Satterstrom et al., 2021). It helps explain why, even in groups with vocal individuals, silence may still dominate—particularly when interpersonal dynamics or group norms discourage dissent (Kim et al., 2023). A group-level approach to voice thus allows researchers and practitioners to better understand the conditions under which voice is encouraged or suppressed across an entire team, and to design interventions that foster inclusive, participatory environments.

Building on this idea, our study is grounded in two primary theoretical perspectives: social network theory and status conflict theory. Drawing on social network theory (Burt, 1992, 2004; Coleman, 1988; M. Granovetter, 1985; Scott, 1991/2000), we argue that the structure of interpersonal relationships within a group—particularly the density of social ties—plays a critical role in shaping group-level outcomes such as group voice behavior. Group network density, defined as the proportion of actual ties to all possible ties in a group, captures the extent of cohesion and redundancy within the social structure. While denser networks are often associated with greater information flow and support, prior research also warns of their potential drawbacks, such as conformity, groupthink, and diminished willingness to express dissenting views (Langfred, 2004; Morrison, 2011). In addition, we incorporate status conflict theory (Bendersky & Hays, 2012; Gould, 2003) to examine how interpersonal struggles for influence within group hierarchies may affect group processes. Status conflict, which emerges when members compete for relative standing and prestige, introduces tensions that can disrupt collaboration and suppress voice behavior. We propose that status conflict—defined as members’ inherent attempts to negotiate and assert their relative standing within the group’s social hierarchy (Bendersky & Hays, 2012)—serves as a critical contextual factor influencing group voice. While group members may appear to maintain harmonious relationships on the surface, such superficial harmony does not eliminate the presence of underlying status dynamics. In fact, tensions related to status hierarchies can still emerge and give rise to sensitive issues. Accordingly, even in groups characterized by close interpersonal ties, latent status conflict may interact with relational structures in complex ways, ultimately shaping the extent and nature of group voice.

By integrating these two frameworks, we aim to capture both the structural and relational dynamics that influence whether—and under what conditions—group members collectively engage in voice. For example, will group harmony serve to encourage group voice? Under what conditions will coworkers tend to believe that voice is benefiting them and under what conditions will they tend to believe otherwise? In turn, the goal of this study is to investigate how group network density and status conflict jointly shape group voice within work teams. Specifically, we aim to uncover the paradoxical conditions under which dense group structures may either encourage or suppress voice, particularly in the presence of status-related tensions. By taking a relational and structural perspective, we contribute to a more nuanced understanding of how group-level social dynamics influence collective voice behaviors.

## 2. Theoretical Background and Hypotheses Development

### 2.1. Group Voice

Voice behavior, broadly defined as discretionary communication intended to improve group or organizational functioning (Van Dyne & LePine, 1998), has often been studied at the individual level. However, our study focuses on group voice, which we define as “the extent to which members of a work group collectively engage in upward communication of suggestions, concerns, or ideas aimed at improving the group’s performance and effectiveness” (Frazier & Bowler, 2015; LePine & Van Dyne, 1998). This collective phenomenon reflects not merely isolated individual behaviors but the shared willingness and joint action of members to speak up as a group. Given that work groups are inherently characterized by interdependence and shared responsibility (Mesmer-Magnus & DeChurch, 2009), voice behavior in the group context is particularly critical, as it can directly contribute to enhanced decision making and more effective identification and resolution of problems (LePine & Van Dyne, 1998; Morrison & Milliken, 2000). Nonetheless, there has been relatively little research for a clearer understanding on the nature of group-level voice behavior.

We conceptualize group voice as a public act of acknowledging group-relevant issues. In contrast to individual voice—which is often a private act of expression—a collective voice emerges when multiple members recognize, validate, and express concerns or suggestions that are widely understood within the group (Zerubavel, 2006). The act of “acknowledging” as a group carries symbolic and social weight, signaling that the issue is not merely a personal concern but one recognized by the group as a whole.

Group voice, defined as the extent to which group members collectively engage in voice behavior, plays a critical role in enabling teams to identify problems, share diverse perspectives, and respond effectively to emerging opportunities (LePine & Van Dyne, 1998). Unlike individual voice, which can be disregarded or dismissed as deviant, group-level voice gains salience and legitimacy when multiple members express similar concerns or suggestions, thereby amplifying its influence on group decisions (Frazier & Bowler, 2015; Yukl & Falbe, 1990). Moreover, because voice behavior affects not only the focal speaker but also their colleagues, it often emerges as a product of collective efforts embedded within group dynamics. Despite its importance, however, research on group voice has largely overlooked its antecedents, particularly those rooted in the group’s relational and structural context. To address this gap, this study seeks to highlight the socially embedded nature of group voice and argues that it is best understood through both relational and structural perspectives.

One compelling yet understudied social phenomenon relevant to group voice is what Zerubavel (2006) terms “the elephant in the room”—a metaphorical expression that captures situations in which all group members are acutely aware of a problem or issue, yet collectively refrain from acknowledging or addressing it. These so-called “open secrets” often persist in team settings, suggesting that voice suppression may not be an individual decision alone, but a relationally conditioned and structurally reinforced outcome (Zettna et al., 2025). This study therefore investigates why group members may choose to remain silent—even when they are metaphorically “in the same room”—and how such silence is shaped by the group’s underlying social network structure. Specifically, we examine group network density, which reflects the degree of interconnectedness among group members, as a key structural condition that may either facilitate or constrain collective voice. At the same time, we explore the role of status conflict, or interpersonal struggles over influence and hierarchical standing, as a relational dynamic that interacts with network structure to suppress or reshape voice behavior. Although group network density and status conflict may appear theoretically incongruent, status-based tensions are often inevitable—even in cohesive groups (Bendersky & Hays, 2012). We propose that attending to the structural dimensions of such conflict enables a more nuanced understanding of the relational constraints that shape whether, and under what conditions, collective voice is expressed or withheld.

### 2.2. The Curvilinear Relationship Between Group Network Density and Group Voice

The literature has suggested that voice behavior is influenced by factors inherent in the social dynamics of the workplace (Brinsfield et al., 2009; Tangirala & Ramanujam, 2008). Despite significant progress made by recent studies in identifying important psychological and personality-based antecedents of voice behavior (Li & Tangirala, 2021; Kim et al., 2023), studies have yet to fully elucidate the influence of social and relational factors at work on such behaviors. Fundamentally, network structures have a significant effect on a group’s ability to potentially absorb or transfer knowledge by voicing their opinions (Tangirala & Ramanujam, 2008). Moreover, since voice impacts others within a network structure, relational and social considerations play an important role in individuals’ decisions about whether to speak up or remain silent (Milliken et al., 2003). These considerations play an integral role, as voice can have both positive and negative implications for other employees in the group. Highlighting a problem, for example, can embarrass other group members; likewise, ideas about potential changes can be a divisive factor within the work group, potentially creating an additional work burden for certain coworkers (LePine & Van Dyne, 1998; Milliken et al., 2003).

Although there are a few studies (for a review, see Venkataramani & Tangirala, 2010) investigating the effects of employees’ network positions on voice behaviors, studies have also not yet fully addressed group-level structural factors such as group density or centralization. In this study, we seek to investigate the relationship between group network density and group voice in particular. Group network density, which refers to the extent to which members in a team are interconnected through social ties, is a key structural measure of a social network (cf. Wasserman & Faust, 1994); thus, it directly indicates the level of redundancy present in a group. Specifically, the network density is reflected by the number of redundant ties existing in the network (Burt, 1992).

Essentially, in order to voice their opinions, group members need to consider two key elements of enablers: the ability and willingness to “take a stand” (Staw & Boettger, 1990). In this regard, social ties, interpersonal connections that link individuals within a network, play an important role in providing the members with abilities to voice because social ties enable a supportive environment, constituting social capital, which facilitates voice behavior at the workplace (M. S. Granovetter, 1973). Social capital refers to the resources embedded in a social network that can be accessed and mobilized through relationships (Nahapiet & Ghoshal, 1998). Therefore, as the number of social ties increases, the number of individuals to whom employees can raise their suggestions increases as well. Accordingly, the effectiveness of spreading and implementing suggestions or ideas will be amplified. In addition, social ties play an important role in enhancing the psychological safety of a group, which is closely tied with the enactment of voice (Detert & Burris, 2007). Thus, with increased social ties within a group, members with high psychological safety are more willing to speak up and readily share ideas and express concerns.

Yet, the social network literature has long suggested the presence of a dark side to group network density. High group network density suggests a tightly connected group where information can flow easily, but it may also lead to conformity and reduced voice behavior. Specifically, previous studies have emphasized that both insufficient and excessive social ties will lead to decreased group performance, for example, in terms of group voice (Langfred, 2004; Wise, 2014). Too little group density, on the one hand, will undermine the group members’ ability to voice to each other because of the lack of accessible social ties. This will reduce the transfer of resources, opportunity, and knowledge conveyed by group voice. Even if members have a true willingness to speak up, they simply do not have access to each other due to a scarcity of interpersonal relationships. In contrast, on the other hand, too much group network density can also reduce group members’ willingness to engage in voice behavior, due to the exceptional efforts required to maintain additional ties, causing the cost to exceed its benefits (Burt, 1992). Research has shown that in order for group members to express their opinions or concerns, they must believe that doing so will be both effective and not too costly personally (Miceli & Near, 1992). In such case, even if they have enough ability to voice, their reduced willingness to take risks will lead to a reduction in their voice behaviors.

Furthermore, due to the fact that voice could upset interpersonal relationships or reflect negatively upon other individuals (LePine & Van Dyne, 1998), employees may decide to refrain from speaking up to avoid harming colleagues or risking a damage to social capital; an individual remaining silent about a coworker’s poor performance can be a good example. Such fears and concerns that members can potentially harm their interpersonal relationships arise when considering whether to convey information about problems, as well as to offer a suggestion for improvement (Detert & Burris, 2007; Milliken et al., 2003). In this regard, too-close relationships among group members may inhibit the way of conveying information about problems or offering a suggestion for improvement because of concerns related to losing their social relationships, such as not wanting to be viewed negatively by others, not wanting to damage a relationship, avoiding upsetting or embarrassing someone else, and fear of retaliation (Dyne et al., 2003; Milliken et al., 2003). As a whole, these factors will reduce the willingness of group members to raise their voice. Taking the aforementioned arguments together, we posit that the positive effects of group network density on group voice may only exist up to a certain level of density, and beyond that, higher density no longer benefits group voice and may even be detrimental. That is, when it exceeds the optimal level, it decreases group voice. Specifically, when density is low to moderate, increasing network density is expected to facilitate greater voice behavior by enhancing access to information and psychological safety. However, when density is moderate to high, further increases in network density are likely to constrain voice due to conformity pressure, coordination burden, and the social costs of deviating from group norms.

**Hypothesis 1.**  *Group network density will have an inverted U-shaped curvilinear relationship with group voice, such that group voice increases at low-to-moderate levels of density but decreases as density moves from moderate to high.*

### 2.3. The Role of Status Conflict in the Relationship Between Group Network Density and Group Voice

Given the influences of structural characteristics (i.e., group network density) on group voice, we further examined the role of interpersonal dynamics in a group to predict group voice. As individuals derive intrinsic value from social esteem (Gould, 2003; Huberman et al., 2004), they are motivated to compete for status while trying to manipulate the social structure of status relations to their advantage (Gould, 2003; Bain et al., 2021). To put it differently, people’s social-structural interests may serve a crucial function, perhaps more important than their task, relationship, or procedural concerns in groups.

Status conflict is defined as disagreements or tension among team members over their relative standing or influence within the group’s hierarchy (Bendersky & Hays, 2012). Unlike task or relationship conflict, status conflict is rooted in competition for prestige and authority, often undermining cooperation, increasing rivalry, and impeding open dialogue and voice. Since status can be regarded as a fixed social resource within a group, gaining one’s status directly results in lowering another member’s rank in the hierarchy, a phenomenon called “zero-sum game” (Berger et al., 1998; Gould, 2003). Therefore, status conflict arises within circumstances wherein members inherently try to control status relations within a group; such structural properties of status conflict, in turn, may result in more competitive behaviors. Previous research on group decision making has revealed that competition, in comparison to cooperation, serves to lessen group information sharing (Toma & Butera, 2009). In particular, voice can be stifled by the salient hierarchy resulted in status conflict. In groups where status competition is salient, voice behavior may be perceived as a threat to others’ standing or as self-promoting, leading members to withhold ideas out of concern for relational costs (Milliken et al., 2003). As such, status conflict creates a relational climate that discourages discretionary communication. Previous research has addressed the fact that the mere introduction of a hierarchical structure into a group tends to inhibit open communication (cf. Festinger et al., 1950). Taken together, we hypothesize that increased competitiveness resulting from status conflict will be detrimental to group voice.

**Hypothesis 2.**  *Status conflict will be negatively associated with group voice.*

In which circumstances do group members tend to believe that voice will be beneficial? LePine and Van Dyne (1998) suggested that the context or situation may have an even more pronounced effect than mere antecedents of voice; situations provide both the stimuli and context, which can be utilized to interpret the stimuli, thus harnessing the potential to impact behavioral responses. Similarly, individual attention shapes their judgments and behaviors (Ocasio, 2011). Likewise, employees constantly seek social cues on whether or not the present work context is favorable for speaking up and utilize these cues to alter their behaviors (Dutton et al., 1997). Thus, organizational contexts have profound impacts on the frequency with which group members speak up.

As indicated above, status conflict is also considered one of the most important contextual factors that affect group processes. Previous research considered conflict as a perceptual rather than behavioral phenomenon (Pruitt & Rubin, 1986). According to this line of research, group members may perceptually interpret contextual status conflict in terms of the way it occurs. That is, as discussed earlier regarding the detrimental effect of excessive group network density, members in extremely cohesive groups are more likely to consider open discussion as threatening the group’s solidarity. As a result, under a high level of status conflict, they tend to make an interpretation of the contextual stimuli as “uncomfortable or unmentionable truths” because they believe that a discretionary voice under high status conflict can be viewed as intended behavior for a particular factional or individual benefit. This is due to the fact that voice has also been classified as a form of “challenging” extra-role behavior which aims at changing the status quo (Van Dyne et al., 1995).

Moreover, highlighting a problem such as status conflict within a group can embarrass others or cast them in a negative light, while ideas for change can create friction within the work group or induce more work for one’s coworkers (LePine & Van Dyne, 1998; Milliken et al., 2003). Accordingly, deliberately withholding useful information often stems from concerns about being labeled as a troublemaker or complainer, or damaging already well-built social capital even if the voice was truly aimed at improving the group’s situation. Indeed, employees may worry about negative repercussions or their credibility (Morrison & Milliken, 2000) when raising an issue, or even making suggestions for improvement. Thus, under high status conflict, as group network density increases, group members are likely to raise their voice, which could undermine the group’s well-established social capital. To summarize, we expect status conflict to moderate the effect of group network density on group voice as follows. When status conflict is high, group network density will be more detrimental than when it is low. Dense networks may actually exacerbate conformity pressures and strategic silence, as members fear that speaking up may harm their standing or create factional tensions (Bendersky & Hays, 2012; Morrison, 2011). Thus, we propose that status conflict moderates the curvilinear relationship between group network density and group voice. Under conditions of high status conflict, the positive slope from low to moderate density becomes flatter, and the negative slope from moderate to high density becomes steeper, resulting in an earlier decline in group voice.

**Hypothesis 3.**  *Status conflict will moderate the inverted U-shaped relationship between group network density and group voice, such that, under high status conflict, (1) the overall level of group voice will be lower, and (2) the curvilinear pattern will become steeper, with group voice decreasing more rapidly beyond the optimal point of density.*

## 3. Method

### 3.1. Research Setting and Data Collection

To test our hypotheses, we analyzed data collected from employees in 55 work groups in three firms in South Korea via a web-based survey. The web-based survey platform allowed for efficient distribution across multiple organizations and ensured anonymity in responses. This method was chosen to minimize potential response bias and enhance respondent comfort when answering sensitive questions about team dynamics, such as interpersonal networks and status conflict (Dillman et al., 2014).

Additionally, online administration enabled broader reach and higher completion rates in organizational settings with limited in-person access due to scheduling constraints. The firms were organized functionally into departments, including engineering, sales, marketing, human resources, and operations. Of 341 questionnaires distributed, 282 were returned, a response rate of 83 percent. Data from one group was excluded because fewer than half of the team members completed the survey, consequently reducing our final sample to 55 groups. The final sample of 282 respondents across 55 work groups was considered sufficient for multilevel analysis. Previous studies suggest that a minimum of 30 groups is generally required to detect group-level effects with acceptable statistical power (Maas & Hox, 2005). Also, the diversity of departments and industries represented in the sample enhances the relevance and generalizability of the findings. The questionnaires included measures of friendship networks among group members, status conflict, and group voice. All respondents were guaranteed confidentiality when they filled out the questionnaires. The average group size was 6.16 (s.d. = 2.93) and average group tenure was 19.48 months (s.d. = 9.38). Of all respondents, 25% were female. The age distribution of respondents indicates that the majority were in their 30s (43.6%), followed by 40s (27.7%), 20s (20.9%), and 50s (7.8%). All respondents had an undergraduate education or higher.

### 3.2. Measurements

Group voice: We measured group voice by estimating group members’ perceptions of group-level voice, and then averaged the workgroup across the members. To avoid common method bias, this study measured group voice by asking team leaders to assess the extent to which their team members actively express opinions on issues that could significantly impact the team. The items that we used are a set of 10 items from Liang et al. (2012). These items consist of promotive and prohibitive voice. The sample items were “As members of our group, the members …”: “…Proactively develop and make suggestions for issues that may influence the unit”, “Raise suggestions to improve the unit’s working procedure”, “Advise other colleagues against undesirable behaviors that would hamper job performance”, and “Dare to voice out opinions on things that might affect efficiency in the work unit, even if that would embarrass others.” All ratings were on a 5-point Likert-type scale ranging from 1 (never) to 5 (very frequently). Cronbach’s alpha for the complete scale was 0.93.

Group network density: To measure network density in group, we employed the roster method in which respondents were asked to check on an alphabetical group member list and check the members of whom they considered “personal friends.” The friendship data from each of the 55 groups were arranged in a separate matrix. Based on employees’ responses, we created a binary matrix of the friendship relations (relation = 1; no relation = 0) among the members, either when they responded themselves or were chosen by others. For each of the 55 groups, we separately calculated the overall density of the friendship network between all members of that group. This measure is the proportion of ties as a function of the total number of possible ties (Wasserman & Faust, 1994). Group density ranges from a minimum of 0 to a maximum of 1. The following Formula (1) was used to calculate network density:

(1) $$Group\;Network\;Density=\;\frac{Actual\;ties}{Total\;number\;of\;possible\;ties}$$

Status conflict: To assess status conflict, we used the 4-item scale from Bendersky and Hays (2012). The items are on a 5-point Likert scale, ranging from 1 (strongly disagree) to 5 (strongly agree). The items included “My team members frequently took sides (i.e., formed coalitions) during conflicts,” “My team members experienced conflicts due to members trying to assert their dominance,” “My team members competed for influence,” and “My team members disagreed about the relative value of members’ contributions.” Cronbach’s alpha for the complete scale was 0.91.

Control variables: We collected a number of control variables. The first was a firm dummy of the companies collected in the sample. This is important since the three companies not only differed in several structural dimensions but also in their management style. Further, these controls were considered to assure that the effect of group density and status conflict can be observed even after any effects of the company-specific factors are controlled.

In addition, following prior research, we controlled for group size by the number of members in a group (Frazier & Bowler, 2015), since group size has implications for a variety of group processes that influence group outcomes (Amason & Sapienza, 1997). Group tenure was also controlled for in the analyses. Training and experience can make group members have more complete cognitive structures and useful knowledge, enabling them to give opinions. We thus controlled for average group tenure (i.e., the mean length of time that group members had been working in the group, measured in months).

A final control variable was psychological safety within a group. This is a multi-item measure which is based on employees’ self-assessments of the level of their group’s psychological safety. According to Detert and Burris (2007), enhanced feelings of psychological safety can foster voice behavior. Thus, we controlled group members’ psychological safety. Members were asked to respond on a 5-point Likert scale, ranging from 1 (strongly disagree) to 5 (strongly agree), to indicate their assessment of the following sample items from Liang et al. (2012) in terms of group psychological safety. The sample items are “In my work unit, I can freely express my thoughts” and “Nobody in my unit will pick on me even if I have different opinions.” Cronbach’s alpha for the complete scale was 0.80.

### 3.3. Data Analyses

We used hierarchical moderated regression analysis at the group level to test our hypotheses. We entered firm dummy variables, group size, group tenure, and psychological safety in step 1, group density and the quadratic group density variable (group density squared) in step 2, status conflict in step 3 and all the independent variables together in step 4, and the interactions in the final step. To assess the curvilinear relationship, we applied a quadratic regression model, as shown in Equation (2).

(2) $$\hat{Y}=\;b_{1}X+\;b_{2}X^{2}+\;b_{3}Z+\;b_{4}XZ+\;b_{5}X^{2}Z+\;b_{0}$$

With hierarchical regression analysis, we can compare alternative models with and without interaction terms. In doing so, we can check a plain interaction effect by separately computing it from the main effects of the independent variables (Jaccard & Turrisi, 2003). The mean-centering process was implemented for the independent variables prior to the formation of interaction terms (Aiken & West, 1991).

For all models, we used several diagnostics of regression to evaluate whether our models were satisfied. First, we checked for the intra-class correlation coefficient, ICC(1) and within-group agreement index, rwg (James et al., 1984), to calculate the appropriateness of aggregating the psychological safety, status conflict, and group voice constructs from our group-level of analysis. ICC(1) is a point estimate of inter-rater reliability in individual-level responses that can be explained by the group level, in which a value greater than 0.12 is generally considered acceptable. The rwg index also justifies group aggregation ranging from 0 to 1, with values greater than 0.70. To be specific, in our data, for psychological safety, the ICC(1) equaled 0.39 and the rwg equaled 0.87; for status conflict, the ICC(1) equaled 0.42 and the rwg equaled 0.85; for group voice, the ICC(1) equaled 0.32 and the rwg equaled 0.97. Thus, the inter-rater reliabilities and the within-group agreement in our analysis were all acceptable.

Next, exploratory factor analysis (EFA) of all the variables revealed three factors with eigenvalues greater than 1.00. Among all items, three items with loading less than 0.50 were dropped, including status conflict (1 item) and psychological safety (2 items). Moreover, in all cases, the three-factor model showed a superior fit to the data (all at the 0.001 level), as demonstrated by the confirmatory factor analysis (CFA). For example, comparing a three-factor model involving psychological safety, status conflict, and group voice to a two-factor model yielded a significant change in chi-square (*p* = 0.001; CFI = 0.80, RMSEA = 0.15). Furthermore, we examined all variance inflation factor (VIF) values and found no multicollinearity concerns (where the highest value obtained in the models was 3.29).

## 4. Results

The correlation is reported in Table 1, which also includes descriptive statistics for all variables in our model. Multicollinearity does not appear to be a problem, because all of the correlations among the variables are below the threshold of 0.7 which is commonly accepted.

Table 2 reports the results of the hierarchical regression analyses when group voice is the dependent variable. In Model 1, we entered the control variables. We then added the group network density and the squared term of group network density in Model 2 to test for the curvilinear relationship proposed in Hypothesis 1. The results indicated that both the first-order and second-order effects of group density became significant. More specifically, the first-order effect was positive (β = 0.85, *p* < 0.001) and the second-order effect turned negative (β = –0.77, *p* < 0.001). The increase in R-square was from 0.06 to 0.32 (F = 9.09, *p* < 0.001). Overall, Model 2 provides support for Hypothesis 1, which proposes that group density exhibits greater group voice when it is at an intermediate (rather than low or high) level of network density.

**Figure 1:**
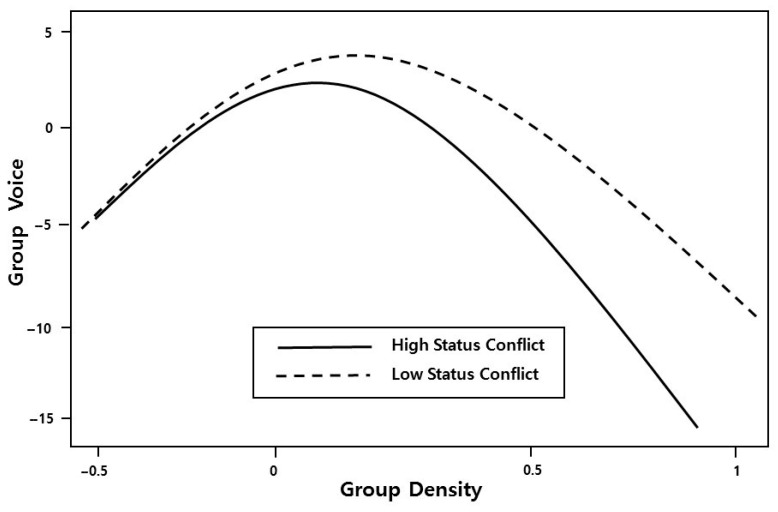
Interaction of group density and status conflict.

Hypothesis 2 is tested in Model 3, where the status conflict variable is introduced. In this model, status conflict is negatively related to group voice (β = −0.38, *p* < 0.05). Thus, Hypothesis 2 is also supported. We also checked all the main effects of group density and status conflict together in Model 4. Finally, Hypothesis 3 was tested in Model 5, where the interaction effect between status conflict and the curvilinear specification of group density was added. The F-test of the increment in R-squared (F = 6.83, *p* < 0.01) indicates that Model 5 is superior to Model 4, such that Model 5 explains 55% of the variation in group voice, and the goodness-of-fit for the whole model is highly satisfactory (an F-value of 5.365, *p* < 0.001). To better illustrate these interaction effects, Figure 1 graphically depicts the curvilinear relationship between group network density and group voice, as well as the moderating effect of status conflict on this relationship.

The curvilinear relationship found between group network density and group voice reveals that network cohesion operates as a double-edged sword. At low-to-moderate levels, increased density appears to foster psychological safety and communication opportunities, thereby encouraging voice. However, from moderate-to-high density, the trend reverses, suggesting that excessive cohesion may create social pressure, inhibit dissent, and discourage constructive expression due to conformity and relational concerns. This pattern aligns with the groupthink literature (Janis, 1982), highlighting that while dense networks enable trust and coordination, they may also suppress divergent thinking and critical voice when overly saturated. Moreover, in highly dense networks, members may feel constrained by expectations of conformity or fear social sanctions for expressing divergent views. Such an inverted U-shaped relationship supports our claim that relational structures do not linearly promote collective outcomes like voice, but rather depend on contextual balance. Furthermore, the interaction effect of status conflict illustrates that this curvilinear pattern is particularly sensitive to the group’s social dynamics. When status conflict is high, the inhibitory effect is amplified—possibly because voice is interpreted as self-interested or status-threatening—indicating that the tipping point of the curve shifts leftward. As a result, even moderate levels of cohesion may be perceived as threatening, thereby accelerating the suppression of voice. These dynamics offer novel insight into how relational strain can recalibrate the effects of structural cohesion.

### Post Hoc Analysis

In order to strengthen our conclusions drawn from the hypotheses test, we re-ran our regression models with dependent variables of two different types of voice behavior: promotive and prohibitive voice, respectively (Liang et al., 2012). Table 3 presents the regression results for promotive and prohibitive group voice, providing further support for our hypotheses. As shown, the curvilinear effect of group network density and the moderating role of status conflict are observed consistently across both types of group voice. Taken together, overall, the proposed models in our study are proven to be robust, as reported in the following Table 3. In sum, these results offer strong empirical support for our hypothesized curvilinear relationship between group network density and group voice (Hypothesis 1). Specifically, we found that group voice is highest at moderate levels of network density, while both very low and very high density levels are associated with reduced voice. This finding aligns with the theoretical notion that moderate connectivity fosters trust and psychological safety, encouraging open communication (Detert & Burris, 2007). In contrast, overly dense networks may foster excessive conformity or fear of social costs, discouraging divergent opinions (Milliken et al., 2003). Furthermore, our analysis supports Hypothesis 2, indicating that status conflict is negatively associated with group voice. This suggests that when group members perceive competition over status, they may become reluctant to express dissenting ideas, viewing such behaviors as risky or politically motivated. These findings are consistent with the prior literature that conceptualizes status conflict as a barrier to open communication and collective information sharing (Bendersky & Hays, 2012; Toma & Butera, 2009). Most importantly, the interaction effects observed in Model 5 provide support for Hypothesis 3, revealing that the negative consequences of high network density are exacerbated under conditions of status conflict. This suggests that in highly cohesive groups, status-related tensions may transform dense networks into politically charged environments where voice is discouraged. In such contexts, voice may be interpreted as a challenge to the social order, leading members to self-censor even in the presence of shared awareness of problems—what Zerubavel (2006) described as the “elephant in the room.” Taken together, these results extend the literature on group voice by highlighting the relational and structural contingencies under which voice behaviors emerge. They also offer practical implications: managers and team leaders should foster a moderate level of group connectedness while actively monitoring and managing status dynamics to ensure that social networks function as enablers—not inhibitors—of collective voice.

## 5. Discussion and Conclusions

This study contributes to the voice behavior literature by examining how group network density and status conflict jointly shape group-level voice. Specifically, social network theory suggests that dense networks facilitate trust, norm enforcement, and resource sharing (Coleman, 1988), all of which can foster collective voice. However, this same tight interconnectivity can also inhibit dissent or unconventional ideas due to conformity pressure, redundancy of information, and risk aversion (Burt, 2004; Langfred, 2004). Thus, we draw on this theoretical tension to propose that the relationship between group network density and group voice follows a curvilinear (inverted U-shaped) pattern, in which moderate levels of density optimize the benefits while avoiding the downsides of overembeddedness.

Complementing this structural view, status conflict theory (Bendersky & Hays, 2012; Gould, 2003) highlights the interpersonal dynamics that arise from competition over relative standing in the group. Status conflicts can reduce cooperation, increase tension, and shape the interpretive context in which group members assess whether speaking up is safe or politically risky. As such, status conflict creates a relational climate that discourages discretionary communication. Specifically, we suggested and found that status conflict is an important boundary condition, and the relation between group density and group voice is moderated when people are exposed to status conflict as a trigger of competitive circumstance. By integrating social network theory with insights from status conflict research, we provide a more comprehensive understanding of the conditions under which voice emerges or is silenced in workgroups. This integration also addresses a theoretical gap in the voice literature, which has traditionally focused on individual-level predictors. A burgeoning portion of the literature theorizes that voice behavior is influenced by social factors inherent in the workplace, the role of group characteristics has not been fully addressed.

Moreover, given the importance of voice in groups (Morrison, 2011), we further develop the literature of voice by creating a model of collective voice, which stands apart from individual-level voice behavior. As indicated above, to voice is rather a public act, so it emerges as a form of collective efforts that involve multiple employees at work (Frazier & Bowler, 2009; Zerubavel, 2006). By conceptualizing voice as a collective phenomenon, we were able to theorize and examine the structural and contextual antecedents of voice behavior (i.e., group network density, status conflict). The current attempt broadens the scope of antecedents of voice behavior, thus facilitating further future research to investigate the role of structural aspects of a group (other than group network density) in predicting group voice.

From a practical standpoint, our results highlight the need for managers to avoid assuming that stronger internal ties always foster openness; in some cases, they may lead to silence due to conformity or political concerns. Managers should be cautious in promoting overly dense networks, especially in hierarchical or status-sensitive teams. Encouraging diverse, cross-cutting ties and establishing norms for inclusive dialogue may help counteract the negative effects of over-cohesion.

Paul Krugman stated that “open secrets constitute uncomfortable truths hidden in plain sight”; such combined behaviors of noticing and ignoring (i.e., withholding voice) arise social phenomena involving more than just individuals. Accordingly, we sought to understand how the structural aspect of a group network (i.e., density) within a group influences collective voice behavior and investigated the relational boundary condition (i.e., status conflict) that may change the implications of key network characteristics.

### Limitations and Future Research

Some of the limitations of this research need to be acknowledged, and they also raise a number of questions for future research. First, due to our cross-sectional data, reverse causality in our model cannot be ruled out. For example, groups whose members are very proactive to speak up may have more social ties. Nonetheless, our arguments posit in the opposite direction because plentiful research evidences that voice is a malleable behavior that is influenced by contextual and social-structural factors (Milliken et al., 2003; Morrison, 2011). Additional research adopting a longitudinal design is needed to verify the direction of causality.

Second, since we tested our framework in firms that were all in South Korea, our findings may not be generalizable to other cultural backgrounds. Indeed, the effect of network structures on employees’ behavior may vary across individualistic or collectivistic cultures. Especially, the social loafing effect, which results from excessively close networks, is more likely to be found in collectivistic cultures such as those of Asian countries, in contrast to individualistic cultures such as the United States and Western European countries (Comer, 1995). Individual group members within a collectivistic culture may lose their motivation due to inappropriate estimation and mismatching efforts (Yang et al., 2015). Thus, such group members may be more likely to withhold their opinions regarding challenging ideas (Kidwell & Bennett, 1993) and less likely to initiate new ideas as a result of excessive group rigidity (Yang et al., 2015). Nevertheless, the interaction effect of group density and voice seems to be culture-free because people, in general, tend to be affected by their embedded social structure with regard to power relations or status rather than just the cultural context. Thus, we encourage future research to test whether our findings are replicable across diverse cultural contexts.

In spite of these limitations, our study is important for future research, as it recognizes the key role of network characteristics in influencing group voice and highlights the importance of structural and relational context factors in voice research. Specifically, we theoretically and empirically expanded the framework for understanding how the structural factors that foster or inhibit group voice relate to one another through an examination of the effects of paradoxical co-existences of group network density and status conflict. Future research could extend these insights by using longitudinal designs or exploring the further role of social and relational context in group voice research.

In sum, this research advances our understanding of group voice by uncovering the paradox that “too much connection can lead to silence”, particularly in environments where status tensions run high. Our model offers a new relational and structural lens for exploring collective silence and opens the door for further research on how group design and social architecture shape communication behavior in teams.

## Figures and Tables

**Table 1:** Correlations and descriptive statistics of variables.

	Mean	SD	1	2	3	4	5	6	7	8
1. Firm Dummy 1	0.26	0.44								
2. Firm Dummy 2	0.31	0.47	−0.391 **							
3. Group Size	6.16	2.93	0.111	0.193						
4. Group Tenure	19.5	9.38	−0.343 *	0.490 **	0.037					
5. Psychological Safety	3.63	0.39	−0.181	0.538 **	0.108	0.330 *				
6. Group Density	0.29	0.17	0.153	0.009	0.001	−0.167	0.327 *			
7. Status Conflict	1.95	0.37	−0.362 **	0.104	0.021	0.293 *	−0.337 *	−0.526 **		
8. Voice	3.50	0.39	−0.113	0.236	0.005	0.104	0.124	0.167	−0.195	

Note: N = 55, * *p* < 0.05, ** *p* < 0.01 (two-tailed test).

**Table 2:** Regression analyses predicting group voice.

Variable	Group Voice				
	Model 1	Model 2	Model 3	Model 4	Model 5
Organization dummy 1	−0.020	−0.070	−0.141	−0.190	−0.200
Organization dummy 2	0.248	0.362 *	0.292	0.411 *	0.449 **
Group Size	−0.039	−0.073	−0.008	−0.047	−0.070
Group Tenure	−0.022	0.097	0.094	0.201	0.149
Psychological Safety	−0.002	−0.207	−0.216	−0.394 *	−0.339 *
Group Density		0.852 ***		0.772 ***	0.839 ***
Group Density squared		−0.774 ***		−0.832 ***	−0.841 ***
Status Conflict			−0.377 *	−0.419 *	−0.477 **
Group Density × Status Conflict					0.476 **
Group Density Squared × Status Conflict					−0.457 **
F	0.605	3.172 **	1.318	3.989 **	5.365 ***
R^2^	0.058	0.321	0.141	0.410	0.549
ΔR^2^	0.058	0.263	0.083	0.352	0.140
ΔF	0.605	9.089 ***	4.659 *	6.914 *	6.827 **

Note: N = 55. * *p* < 0.05, ** *p* < 0.01, *** *p* < 0.001 (two-tailed test). Standardized regression coefficients (β).

**Table 3:** Regression analyses predicting two types of group voice.

Variable	Promotive Group Voice					Prohibitive Group Voice				
	Model 1	Model 2	Model 3	Model 4	Model 5	Model 1	Model 2	Model 3	Model 4	Model 5
Firm Dummy 1	0.253	0.210	0.115	0.080	0.079	−0.260 †	−0.309 *	−0.347 *	−0.398 **	−0.416 **
Firm Dummy 2	0.287	0.371 *	0.337 †	0.424 *	0.474 **	0.172	0.294 †	0.204	0.330 *	0.352 *
Group Size	−0.173	−0.195	−0.137	−0.166	−0.181	0.087	0.048	0.110	0.068	0.041
Group Tenure	0.043	0.141	0.175	0.254 †	0.181	−0.077	0.042	0.006	0.120	0.096
Psychological Safety	0.031	−0.139	−0.212	−0.343 †	−0.285 †	−0.031	−0.233	−0.184	−0.374 *	−0.331 *
Group Density		0.656 **		0.569 **	0.610 **		0.884 ***		0.824 ***	0.903 ***
Group Density Square		−0.548 **		−0.612 **	−0.612 **		−0.845 ***		−0.889 ***	−0.905 ***
Status Conflict			−0.426 *	−0.457 **	−0.514 **			−0.269	−0.315 *	−0.363 *
Group Density × Status Conflict					0.397 *					0.466 **
Group Density Square × Status Conflict					−0.452 **					−0.384 **
F	1.137	2.242 *	2.136 †	3.176 **	3.921 **	1.241	4.680 ***	1.467	4.914 ***	6.166 ***
R^2^	0.104	0.250	0.211	0.356	0.471	0.112	0.411	0.155	0.461	0.584
ΔR^2^	0.104	0.146	0.107	0.252	0.115	0.112	0.298	0.043	0.050	0.123
ΔF	1.137	4.586 *	6.491 *	7.534 **	4.800 *	1.241	11.896 ***	2.415	4.273 *	6.485 **

Note: N = 55. † *p* < 0.10, * *p* < 0.05, ** *p* < 0.01, *** *p* < 0.001 (two-tailed test). Standardized regression coefficients (β).

## Data Availability

The data of this study are available from the corresponding author upon reasonable request.

## References

[B1-behavsci-15-00926] Aiken L., West S. (1991). Multiple regression: Testing and interpreting interactions.

[B2-behavsci-15-00926] Amason A. C., Sapienza H. J. (1997). The effects of top management team size and interaction norms on cognitive and affective conflict. Journal of Management.

[B3-behavsci-15-00926] Bain K., Kreps T. A., Meikle N. L., Tenney E. R. (2021). Amplifying voice in organizations. Academy of Management Journal.

[B4-behavsci-15-00926] Bendersky C., Hays N. A. (2012). Status conflict in groups. Organization Science.

[B5-behavsci-15-00926] Berger J., Ridgeway C. L., Fisek M. H., Norman R. Z. (1998). The legitimation and delegitimation of power and prestige orders. American Sociological Review.

[B6-behavsci-15-00926] Brinsfield C. T., Edwards M. S., Greenberg J. (2009). Voice and silence in organizations: Historical review and current conceptualizations.

[B7-behavsci-15-00926] Burt R. S. (1992). Structural holes: The social structure of competition.

[B8-behavsci-15-00926] Burt R. S. (2004). Structural holes and good ideas. American Journal of Sociology.

[B9-behavsci-15-00926] Coleman J. S. (1988). Social capital in the creation of human capital. American Journal of Sociology.

[B10-behavsci-15-00926] Comer D. R. (1995). A model of social loafing in real work groups. Human Relations.

[B11-behavsci-15-00926] De Dreu C. K., West M. A. (2001). Minority dissent and team innovation: The importance of participation in decision making. Journal of Applied Psychology.

[B12-behavsci-15-00926] Detert J. R., Burris E. R. (2007). Leadership behavior and employee voice: Is the door really open?. Academy of Management Journal.

[B13-behavsci-15-00926] Dillman D. A., Smyth J. D., Christian L. M. (2014). Internet, phone, mail, and mixed-mode surveys: The tailored design method.

[B14-behavsci-15-00926] Dutton J. E., Ashford S. J., O’neill R. M., Hayes E., Wierba E. E. (1997). Reading the wind: How middle managers assess the context for selling issues to top managers. Strategic Management Journal.

[B15-behavsci-15-00926] Dyne L. V., Ang S., Botero I. C. (2003). Conceptualizing employee silence and employee voice as multidimensional constructs. Journal of Management Studies.

[B16-behavsci-15-00926] Festinger L., Schachter S., Back K. (1950). Social pressures in informal groups, a study of human factors in housing.

[B17-behavsci-15-00926] Frazier M. L., Bowler W. M. (2009). Voice climate in organizations: A group-level examination of antecedents and performance outcomes. Annual Meeting of the Academy of Management.

[B18-behavsci-15-00926] Frazier M. L., Bowler W. M. (2015). Voice climate, supervisor undermining, and work outcomes: A group-level examination. Journal of Management.

[B19-behavsci-15-00926] Gould R. V. (2003). Collision of wills: How ambiguity about social rank breeds conflict.

[B20-behavsci-15-00926] Granovetter M. (1985). Economic action and social structure: The problem of embeddedness. American Journal of Sociology.

[B21-behavsci-15-00926] Granovetter M. S. (1973). The strength of weak ties. American Journal of Sociology.

[B22-behavsci-15-00926] Huberman B. A., Loch C. H., Önçüler A. (2004). Status as a valued resource. Social Psychology Quarterly.

[B23-behavsci-15-00926] Jaccard J., Turrisi R. (2003). Interaction effects in multiple regression.

[B24-behavsci-15-00926] James L. R., Demaree R. G., Wolf G. (1984). Estimating within-group interrater reliability with and without response bias. Journal of Applied Psychology.

[B25-behavsci-15-00926] Janis I. L. (1982). Groupthink: Psychological studies of policy decisions and fiascoes.

[B26-behavsci-15-00926] Kidwell R. E., Bennett N. (1993). Employee propensity to withhold effort: A conceptual model to intersect three avenues of research. Academy of Management Review.

[B27-behavsci-15-00926] Kim Y. J., Lam C. F., Oh J., Sohn W. (2023). Employee constructive voice: An integrative review and a dyadic approach. Journal of Management.

[B28-behavsci-15-00926] Langfred C. W. (2004). Too much of a good thing? Negative effects of high trust and individual autonomy in self-managing teams. Academy of Management Journal.

[B29-behavsci-15-00926] LePine J. A., Van Dyne L. (1998). Predicting voice behavior in work groups. Journal of Applied Psychology.

[B30-behavsci-15-00926] LePine J. A., Van Dyne L. (2001). Voice and cooperative behavior as contrasting forms of contextual performance: Evidence of differential relationships with Big Five personality characteristics and cognitive ability. Journal of Applied Psychology.

[B31-behavsci-15-00926] Li A. N., Liao H., Tangirala S., Firth B. M. (2017). The content of the message matters: The differential effects of promotive and prohibitive team voice on team productivity and safety performance gains. Journal of Applied Psychology.

[B32-behavsci-15-00926] Li A. N., Tangirala S. (2021). How voice emerges and develops in newly formed supervisor–employee dyads. Academy of Management Journal.

[B33-behavsci-15-00926] Liang J., Farh C. I., Farh J. L. (2012). Psychological antecedents of promotive and prohibitive voice: A two-wave examination. Academy of Management Journal.

[B34-behavsci-15-00926] Maas C. J. M., Hox J. J. (2005). Sufficient sample sizes for multilevel modeling. Methodology: European Journal of Research Methods for the Behavioral and Social Sciences.

[B35-behavsci-15-00926] Mesmer-Magnus J. R., DeChurch L. A. (2009). Information sharing and team performance: A meta-analysis. Journal of Applied Psychology.

[B36-behavsci-15-00926] Miceli M. P., Near J. P. (1992). Blowing the whistle: The organizational and legal implications for companies and employees.

[B37-behavsci-15-00926] Milliken F. J., Morrison E. W., Hewlin P. F. (2003). An exploratory study of employee silence: Issues that employees don’t communicate upward and why. Journal of Management Studies.

[B38-behavsci-15-00926] Morrison E. W. (2011). Employee voice behavior: Integration and directions for future research. Academy of Management Annals.

[B39-behavsci-15-00926] Morrison E. W., Milliken F. J. (2000). Organizational silence: A barrier to change and development in a pluralistic world. Academy of Management Review.

[B40-behavsci-15-00926] Nahapiet J., Ghoshal S. (1998). Social capital, intellectual capital, and the organizational advantage. Academy of Management Review.

[B41-behavsci-15-00926] Ocasio W. (2011). Attention to attention. Organization Science.

[B42-behavsci-15-00926] Pruitt D. G., Rubin J. Z. (1986). Social conflict: Escalation, impasse, and resolution.

[B43-behavsci-15-00926] Satterstrom P., Kerrissey M., DiBenigno J. (2021). The voice cultivation process: How team members can help upward voice live on to implementation. Administrative Science Quarterly.

[B44-behavsci-15-00926] Scott J. (2000). Social network analysis: A handbook.

[B45-behavsci-15-00926] Sherf E. N., Sinha R., Tangirala S., Awasty N. (2018). Centralization of member voice in teams: Its effects on expertise utilization and team performance. Journal of Applied Psychology.

[B46-behavsci-15-00926] Staw B. M., Boettger R. D. (1990). Task revision: A neglected form of work performance. Academy of Management Journal.

[B47-behavsci-15-00926] Tangirala S., Ramanujam R. (2008). Employee silence on critical work issues: The cross-level effects of procedural justice climate. Personnel Psychology.

[B48-behavsci-15-00926] Toma C., Butera F. (2009). Hidden profiles and concealed information: Strategic information sharing and use in group decision making. Personality and Social Psychology Bulletin.

[B49-behavsci-15-00926] Van Dyne L., Cummings L. L., Parks J. M. (1995). Extra-role behaviors-in pursuit of construct and definitional clarity (a bridge over muddied waters). Research in Organizational Behavior: An Annual Series of Analytical Essays and Critical Reviews.

[B50-behavsci-15-00926] Van Dyne L., LePine J. A. (1998). Helping and voice extra-role behaviors: Evidence of construct and predictive validity. Academy of Management Journal.

[B51-behavsci-15-00926] Venkataramani V., Tangirala S. (2010). When and why do central employees speak up? An examination of mediating and moderating variables. Journal of Applied Psychology.

[B52-behavsci-15-00926] Wasserman S., Faust K. (1994). Social network analysis: Methods and applications.

[B53-behavsci-15-00926] Wise S. (2014). Can a team have too much cohesion? The dark side to network density. European Management Journal.

[B54-behavsci-15-00926] Yang Z., Zhou X., Zhang P. (2015). Discipline versus passion: Collectivism, centralization, and ambidextrous innovation. Asia Pacific Journal of Management.

[B55-behavsci-15-00926] Yukl G., Falbe C. M. (1990). Influence tactics and objectives in upward, downward, and lateral influence attempts. Journal of Applied Psychology.

[B56-behavsci-15-00926] Zerubavel E. (2006). The elephant in the room: Silence and denial in everyday life.

[B57-behavsci-15-00926] Zettna N., Nguyen H., Restubog S. L. D., Schilpzand P., Johnson A. (2025). How teams can overcome silence: The roles of humble leadership and team commitment. Personnel Psychology.

[B58-behavsci-15-00926] Zheng X., Liu X., Liao H., Qin X., Ni D. (2022). How and when top manager authentic leadership influences team voice: A moderated mediation model. Journal of Business Research.

